# Atrial Fibrillation in an Unroofed Coronary Sinus

**DOI:** 10.1016/j.case.2026.02.007

**Published:** 2026-04-09

**Authors:** Jessica Victoria Yao, Kai' En Leong

**Affiliations:** a Department of Cardiology, Royal Melbourne Hospital, Parkville, Victoria, Australia; b Department of Medicine, Dentistry and Health Sciences, University of Melbourne, Parkville, Victoria, Australia

**Keywords:** Atrial fibrillation, Unroofed coronary sinus, Congenital heart disease, ACHD, Multimodal imaging

## Abstract

• AF in young adults should prompt investigation of structural heart disease.
• Unroofed CS is a very rare abnormality accounting for <1% of all ASDs.
• Unroofed CS is difficult to diagnose on TTE.
• TEE, CCT, or CMR should be considered in patients with unexplained RV dilatation.
• AF in unroofed CS is postulated to arise from the CS ostium.

## Graphical abstract

**Figure:**
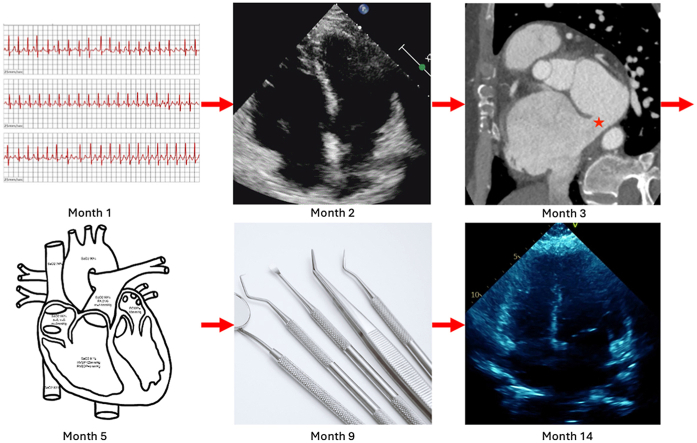

## Introduction

Atrial fibrillation (AF) is less common in middle adulthood.^1^ Diagnosis should prompt investigation for underlying causes such as cardiomyopathy, valvular heart disease, or structural heart disease. Family history should also be considered.^2^ In those below the age of 45, without structural abnormalities, genetic testing should also be considered.^1^

## Case Presentation

A 43-year-old nulliparous female patient of Japanese descent presented to clinic with intermittent palpitations. The palpitations were fast and irregular, lasting seconds with no associated presyncope or syncope. The episodes occurred every few weeks with no obvious precipitant. Examination was unremarkable. Body mass index was normal at 23.

Past medical history included endometriosis. Family history was unremarkable for cardiac disease. They did not smoke and consumed alcohol socially. The patient was active but did not engage in endurance sports.

On examination, blood pressure was 110/80 mm Hg, heart rate was 80 bpm, and oxygen saturation was 99% on room air. Heart sounds were dual with no audible murmurs, and chest was clear. Electrocardiogram demonstrated sinus rhythm, right-axis deviation, and a premature atrial contraction. Given infrequent symptoms, a 28 day ambulatory electrocardiogram was performed. This demonstrated sinus rhythm with occasional atrial ectopy, atrial couplets, and nonsustained atrial runs lasting up to 27 seconds. It also showed occasional AF and atrial flutter with intermittent rapid ventricular response up to 194 beats per minute.

**Figure 1:**
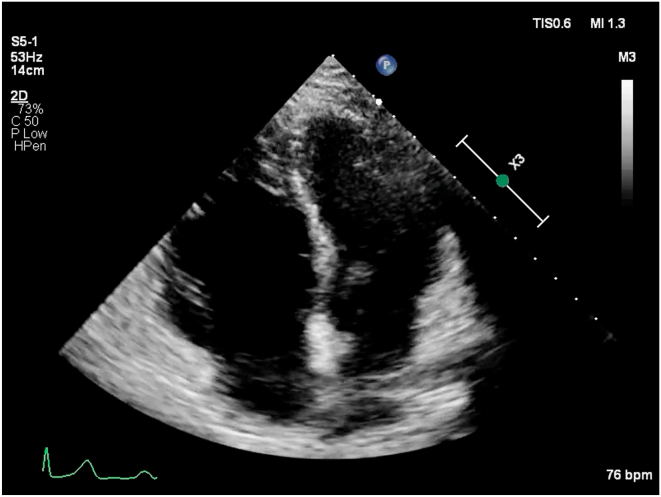
Two-dimensional TTE, apical 4-chamber diastolic view, demonstrates a dilated RV and RA with abnormal septal position consistent with right ventricular volume overload.

**Figure 2:**
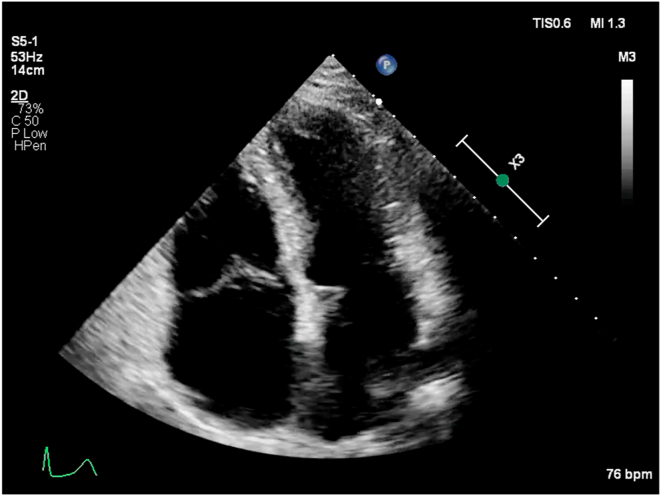
Two-dimensional TTE, apical 4-chamber systolic view, demonstrates a dilated RV and RA with abnormal septal position consistent with right ventricular volume overload.

**Figure 3:**
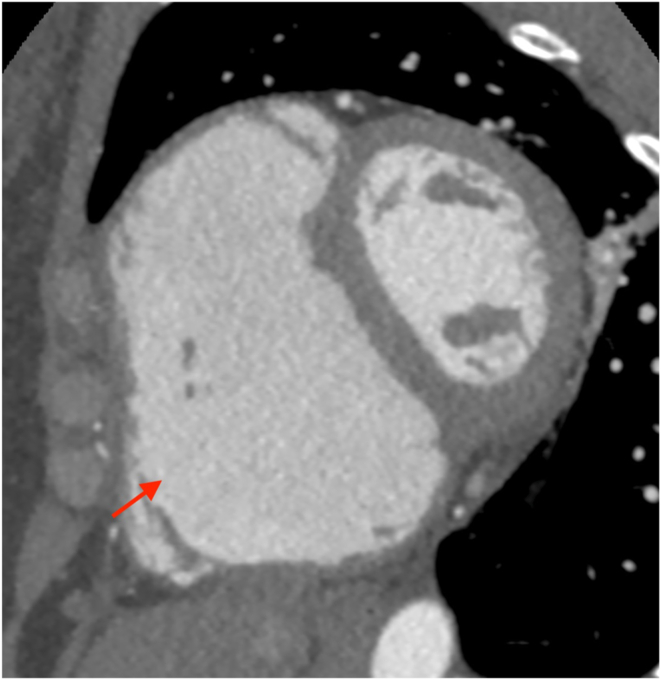
Cardiac computed tomography, oblique sagittal plane, cardiac mid-LV short-axis view, demonstrates a dilated RV *(arrow)* with abnormal septal position consistent with right ventricular volume-overload.

**Figure 4:**
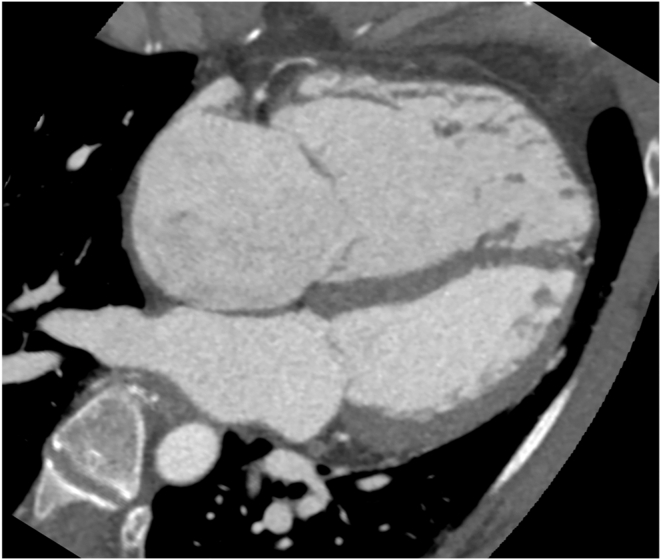
Cardiac computed tomography, oblique axial plane, cardiac 4-chamber view, demonstrates a dilated RV with abnormal septal position consistent with right ventricular volume overload.

**Figure 5:**
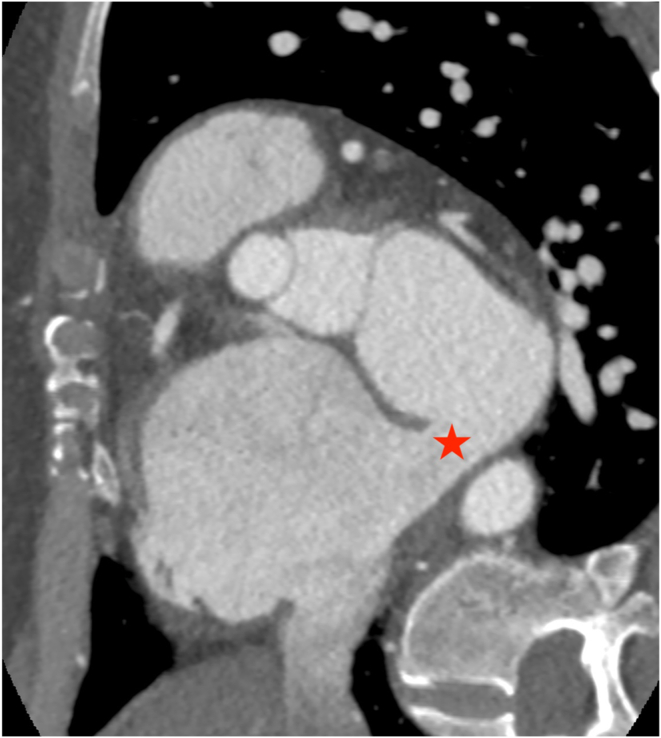
Cardiac computed tomography, oblique sagittal plane, cardiac basal-LV short-axis view demonstrates a completely unroofed CS with an unrestricted LA-CS communication *(star)*.

**Figure 6:**
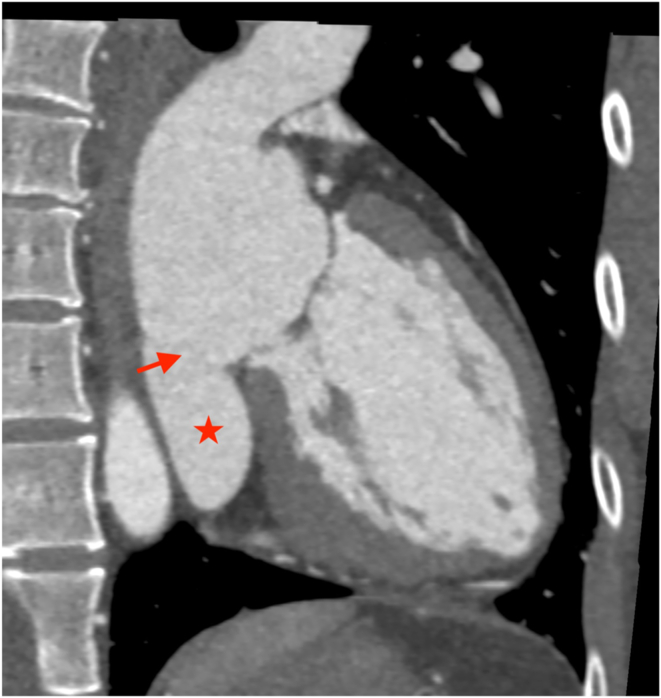
Cardiac computed tomography, oblique sagittal plane, cardiac 2-chamber view, demonstrates the absence of inferior LA “floor” *(arrow)* with LA-CS communication and severely dilated CS *(star)*.

**Figure 7:**
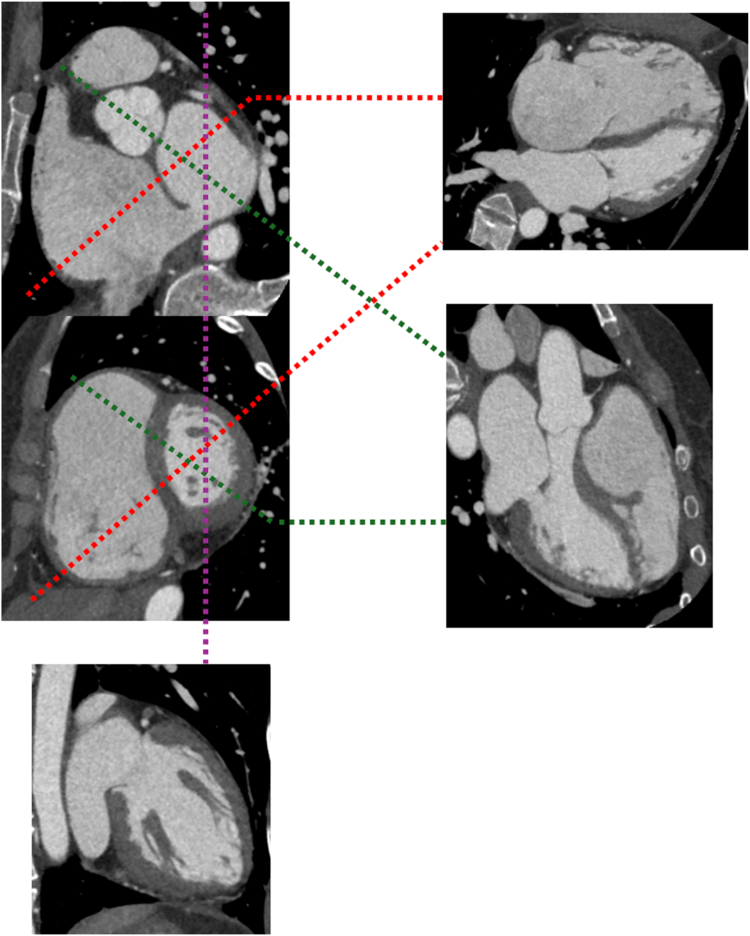
Cardiac computed tomography in multiple different planes (*red line*: apical 4-chamber; *green line*: apical 3-chamber; *purple line*: apical 2-chamber) demonstrates that the 2-chamber view best demonstrates the unroofed CS.

**Figure 8:**
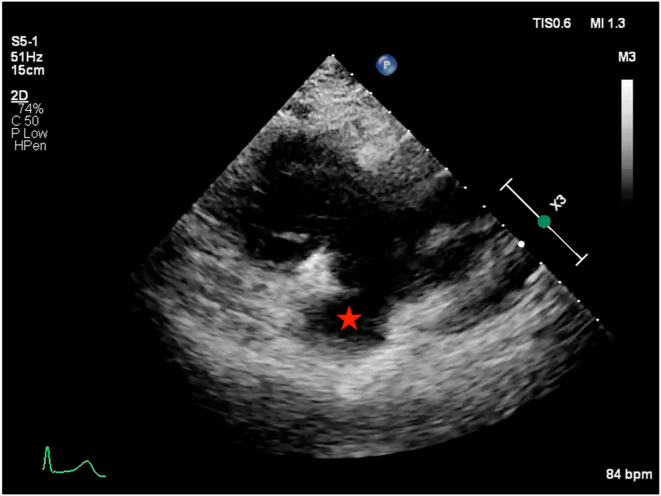
Two-dimensional TTE, parasternal right ventricular inflow view, demonstrates a dilated right heart with dilated CS *(star)*.

**Figure 9:**
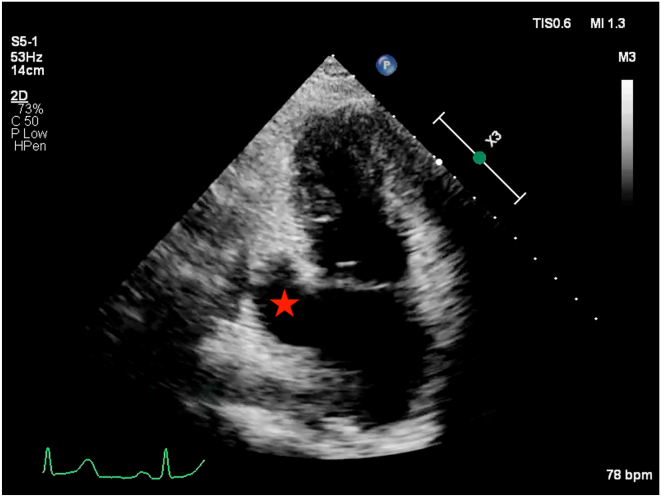
Two-dimensional TTE, apical 2-chamber systolic view, demonstrates the dilated CS with a deficient LA floor *(star)*.

**Figure 10:**
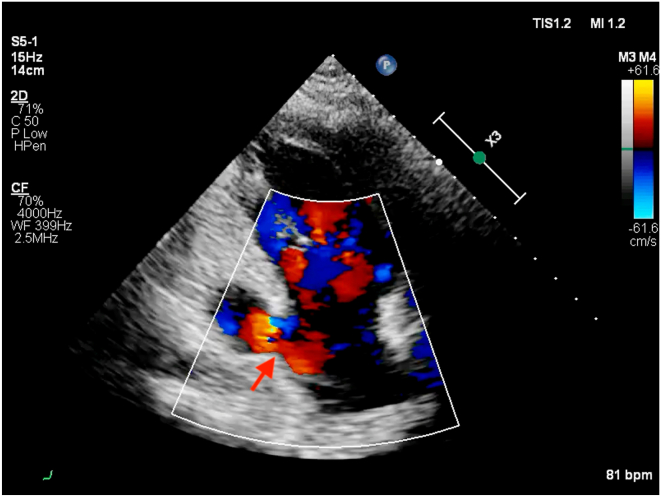
Two-dimensional TTE, apical 2-chamber systolic view with color-flow Doppler, demonstrates the dilated CS with a deficient LA floor *(star)* and laminar flow connecting these chambers *(arrow)*.

**Figure 11:**
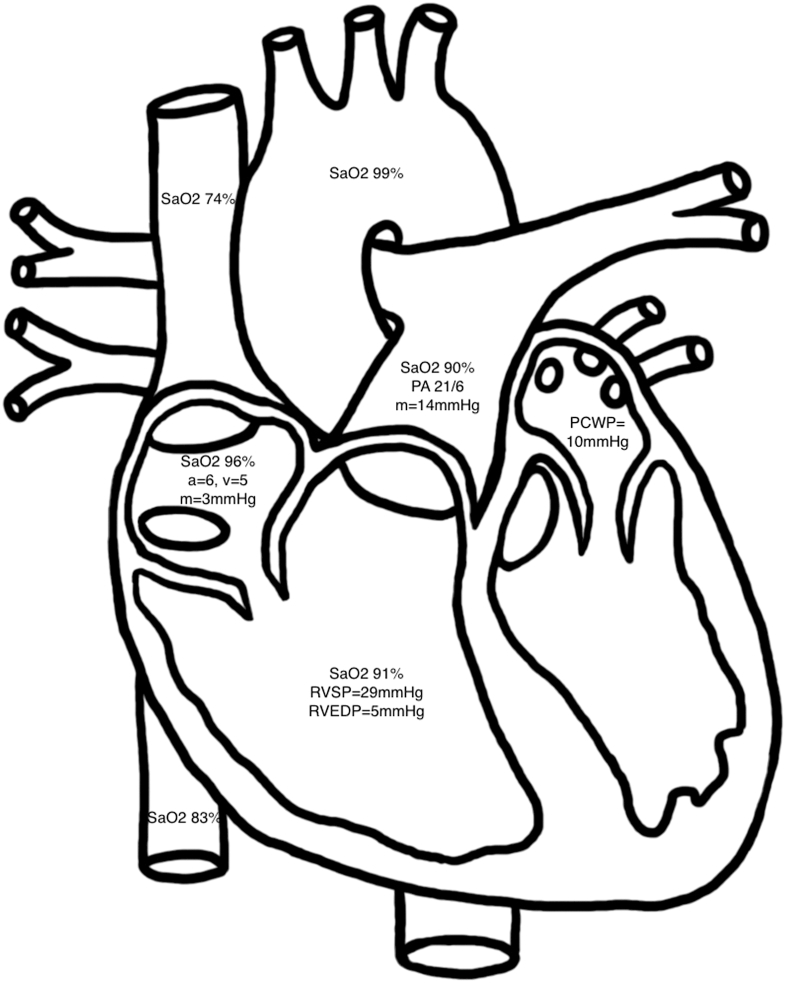
Schematic representation of the hemodynamic findings during right heart catheterization, including oxygenation levels in each of the cardiac chambers, demonstrates the left-to-right shunt-induced step-up.

Transthoracic echocardiogram (TTE) demonstrated normal left ventricular size and systolic function but a severely enlarged right ventricle (RV; right ventricular base, 5 cm) with preserved systolic function (tricuspid annular plane systolic excursion 20, right ventricular S′ 11; Figures 1 and 2, Videos 1-3). The right atrium (RA) was also enlarged, but pulmonary pressures were normal. A shunt was not appreciated on this initial study. Considering these findings, cardiac computed tomography (CCT) was performed (Figure 3, Figure 4, Figure 5, Figure 6, Figure 7, Videos 4-9), revealing a completely unroofed coronary sinus (CS) with left-to-right shunting. The CS was severely dilated. The ostium measured 19 × 36 mm. The RA (4-chamber right atrial area 29 cm^2^ vs left atrial area 20 cm^2^), RV (4-chamber RV: right ventricular basal dimension ratio >2), and main pulmonary artery (28 mm, pulmonary artery: aorta ratio >1) were also severely dilated. Pulmonary venous drainage was normal, and there was a single right-sided superior vena cava. A repeat dedicated TTE also demonstrated a deficient left atrial “floor.” Color-flow Doppler confirmed flow from the left atrium to the CS (Figure 8, Figure 9, Figure 10, Videos 10-12). Right heart catheterization demonstrated a significant left-to-right shunt with a Qp:Qs of 2.5:1 and normal pulmonary artery pressures (mean gradient, 14 mm Hg; Figure 11). Oxygen saturation increased significantly in the RA (SaO_2_ 96%) compared to the superior vena cava (SaO_2_ 74%) and inferior vena cava (SaO_2_ 83%), consistent with a shunt at the atrial level. An electrophysiology study was considered. However, previous reports have postulated that in patients with an unroofed CS, AF arises from the ostium of the dilated CS. Thus, it was felt that surgical correction of the unroofed CS might mitigate AF. The patient proceeded to have successful surgery through a median sternotomy. The RA was opened obliquely, and the interatrial septum was exposed. The large CS defect was seen and measured approximately 2.0 cm × 2.0 cm. A fresh patch of autologous pericardium was used to close the defect. The left atrial appendage was then closed with a 35 mm clip. A MAZE procedure was considered, but the patient elected not to proceed with this given the risk of complete heart block. Five months postsurgery, the patient remains asymptomatic with no further palpitations. Ambulatory event monitor demonstrated no further episodes of AF; TTE demonstrated normal biventricular and biatrial size.

## Discussion

Unroofed CS is a rare congenital abnormality, accounting for <1% of all atrial septal defects.^3^ Unroofed CS was first described in 1965 and is characterized by rupture or complete absence of the CS wall, resulting in communication between the CS and left and right atria.^4^ In most cases, unroofed CS is associated with other congenital heart defects; 75% are associated with a persistent left-sided superior vena cava (LSVC).^4^ Other associations include mitral, pulmonary, or tricuspid atresia, atrial isomerism, atrioventricular septal defect, mitral stenosis, and tetralogy of Fallot.^3^^,^^4^ There are 4 types of unroofed CS. Type 1 is characterized by a completely unroofed CS with an LSVC. Type 2 is defined by a completely unroofed CS without an LSVC. Type 3 is characterized by a partially unroofed midportion, and type 4 by a partially unroofed terminal portion. Isolated unroofed CS (type 2), as in our case, is extremely rare.

Clinical manifestations are nonspecific and can include exertional dyspnea, fatigue, and signs of heart failure. Syncope, peripheral edema, and recurrent respiratory infections have also been described.^4^ Atrial fibrillation tends to occur in older patients with unroofed CS.^5^ Only a few cases of AF in unroofed CS have been described in the literature. Tsuji *et al.*^5^ describe a case of AF in a 74-year-old woman who was found to have an unroofed CS. This was successfully managed with a pulmonary vein isolation and CS ablation. Miyashita and Suzuki^6^ describe a 75-year-old man with AF and mitral regurgitation, who was found to have an unroofed CS. In this case, the CS defect was surgically repaired. Watanabe *et al.*^7^ describe a 67-year-old woman who presented with AF and was also found to have an unroofed CS. Nishimura *et al.*^8^ describe a similar case in a 55-year-old man who was treated with surgery. Interestingly, all patients were of Japanese descent. We describe the youngest case of AF in an unroofed CS.

The exact mechanism of AF in patients with an unroofed CS remains elusive. Previous case reports have postulated that AF arises from the ostium of the dilated CS, rather than the pulmonary veins. Tsuji *et al.*^5^ describe sustained AF due to premature contractions from the CS ostium after wide circumferential pulmonary vein isolation. Upon ablation of the ostial region of the CS floor, AF was no longer inducible. Furthermore, in patients who have developed recurrent AF after unroofed CS repair and concomitant MAZE procedure, CS ablation has been shown to be effective.

Diagnosis of unroofed CS can be challenging, particularly when coexistent with another cyanotic cardiac malformation.^9^ A large number of unroofed CSs are still diagnosed intraoperatively.^10^ Unroofed CS can be diagnosed on TTE but is often missed due to its posterior location, as in our case initially. Cardiac computed tomography has superior temporal and spatial resolution and can better evaluate the venous system and extracardiac structures. Cardiac computed tomography also allows three-dimensional visualization of anatomy.^9^ Other imaging modalities include transesophageal echocardiography (TEE) and cardiovascular magnetic resonance imaging (CMR). Transesophageal echocardiography is particularly helpful for identifying posterior structures and has superior spatial resolution.^10^ Transesophageal echocardiography is also helpful for determining direction of blood flow without the need for contrast.^11^ Three-dimensional TEE has been found to further enhance accuracy in delineating anatomical relationships with adjacent structures.^12^ A TEE was organized in our case but did not eventuate given the diagnosis was already established on CCT. Cardiovascular magnetic resonance imaging allows visualization of all cardiac and extracardiac manifestations and can also be used to determine functional information. Right heart catheterization can be used to confirm presence of the defect and to determine the Qp:Qs ratio. Surgical patch repair is the mainstay treatment of unroofed CS.^10^ Transcatheter closure using an Amplatzer septal occluder or covered stent has also been described as a treatment option in type 2 and 3 unroofed CS with adequate rims.^4^^,^^10^

## Conclusion

Unroofed CS is a rare congenital abnormality. Diagnosis should be considered in patients with an unexplained dilated RV. Atrial fibrillation in unroofed CS typically occurs with right heart dilatation and has been postulated to arise from the ostium of the CS rather than the pulmonary veins. Cardiac computed tomography, CMR, and/or TEE are helpful for diagnosing unroofed CS due to the posterior location. Mainstay treatment for unroofed CS is surgery. Pulmonary vein isolation may not be effective in these patients.

## Ethics Statement

The authors declare that the work described has been carried out in accordance with The Code of Ethics of the World Medical Association (Declaration of Helsinki) for experiments involving humans.

## Consent Statement

Complete written informed consent was obtained from the patient (or appropriate parent, guardian, or power of attorney) for the publication of this study and accompanying images.

## Funding

The authors declare that this report did not receive any specific grant from funding agencies in the public, commercial, or not-for-profit sectors.

## Disclosure Statement

The authors reported no actual or potential conflicts of interest relative to this document.

## Supplementary Data

Video 1 Two-dimensional TTE, apical 4-chamber view, demonstrates a dilated RV and RA with abnormal septal position consistent with right ventricular volume overload.

Video 2 Two-dimensional TTE, parasternal long-axis view, demonstrates a dilated RV with abnormal septal position consistent with right ventricular volume overload.

Video 3 Two-dimensional TTE, parasternal long-axis view, demonstrates a dilated RV with abnormal septal position consistent with right ventricular volume overload.

Video 4 Cardiac computed tomography, axial stack, superior to inferior, demonstrates the dilated right heart with completely unroofed CS.

Video 5 Cardiac computed tomography, sagittal stack, right to left, demonstrates the dilated right heart with completely unroofed CS.

Video 6 Two-dimensional TTE, parasternal right ventricular inflow view, demonstrates a dilated right heart with dilated CS.

Video 7 Two-dimensional TTE, apical 2-chamber view, demonstrates normal left ventricular size and systolic function with the dilated CS with a deficient LA floor.

Video 8 Two-dimensional TTE, apical 2-chamber view with color-flow Doppler, demonstrates the dilated CS with a deficient LA floor and laminar flow connecting these chambers.

## References

[1] Joglar J.A., Chung M.K., Armbruster A.L., Benjamin E.J., Chyou J.Y., Cronin E.M. (2024). 2023 ACC/AHA/ACCP/HRS Guideline for the diagnosis and management of atrial fibrillation: a report of the American College of Cardiology/American Heart Association Joint Committee on Clinical Practice Guidelines. J Am Coll Cardiol.

[2] Van Gelder I.C., Rienstra M., Bunting K.V., Casado-Arroyo R., Caso V., Crijns H. (2024). 2024 ESC Guidelines for the management of atrial fibrillation developed in collaboration with the European Association for Cardio-Thoracic Surgery (EACTS). Eur Heart J.

[3] Murli L., Ranjit M.S., Shah P. (2019). Unroofed coronary sinus: an unusual interatrial communication and a rare childhood entity. Ann Pediatr Cardiol.

[4] Cinteză E.E., Filip C., Duică G., Nicolae G., Nicolescu A.M., Bălgrădean M. (2019). Unroofed coronary sinus: update on diagnosis and treatment. Rom J Morphol Embryol.

[5] Tsuji M., Kato K., Tanaka H., Tejima T. (2017). Pulmonary vein isolation for paroxysmal atrial fibrillation in a patient with stand-alone unroofed coronary sinus. HeartRhythm Case Rep.

[6] Miyashita F., Suzuki T. (2024). Surgical repair of a coronary sinus atrial septal defect in an elderly patient. Ann Thorac Surg Short Rep.

[7] Watanabe N., Yanagita Y., Matsuura H., Nishino S., Nishimura M., Yano M. (2016). Unroofed coronary sinus detected by 2D/3D echocardiography in a patient referred to catheter ablation for atrial fibrillation. J Cardiol Cases.

[8] Nishimura O., Ueyama K., Nagayoshi Y., Ueyama T. (2014). [Surgical treatment of unroofed coronary sinus coexisting with paroxysmal atrial fibrillation; diagnosis by multidetector computed tomography; report of a case]. Kyobu Geka.

[9] Ma J., Zheng Y., Xu S., Teng H., Lv L., Li Y. (2022). The value of cardiac CT in the diagnosis of unroofed coronary sinus syndrome. BMC Cardiovasc Disord.

[10] Slingerland A., Moolla M., John K., Dennett L., Nagendran J., Mathew A. (2025). Invasive management of unroofed coronary sinus: a systematic review. Trends Cardiovasc Med.

[11] Sun T., Fei H.W., Huang H.L., Chen O.D., Zheng Z.C., Zhang C.J. (2014). Transesophageal echocardiography for coronary sinus imaging in partially unroofed coronary sinus. Echocardiography.

[12] Johri A.M., Witzke C., Solis J., Palacios I.F., Inglessis I., Picard M.H. (2011). Real-time three-dimensional transesophageal echocardiography in patients with secundum atrial septal defects: outcomes following transcatheter closure. J Am Soc Echocardiogr.

